# Cytotoxicity of replication-competent adenoviruses powered by an exogenous regulatory region is not linearly correlated with the viral infectivity/gene expression or with the E1A-activating ability but is associated with the *p53* genotypes

**DOI:** 10.1186/s12885-017-3621-x

**Published:** 2017-09-05

**Authors:** Suguru Yamauchi, Boya Zhong, Kiyoko Kawamura, Shan Yang, Shuji Kubo, Masato Shingyoji, Ikuo Sekine, Yuji Tada, Koichiro Tatsumi, Hideaki Shimada, Kenzo Hiroshima, Masatoshi Tagawa

**Affiliations:** 1 Division of Pathology and Cell Therapy, Chiba Cancer Center Research Institute, Chiba, Japan; 2 Department of Molecular Biology and Oncology, Graduate School of Medicine, Chiba University, Chiba, Japan; 3 Department of Genetics, Hyogo College of Medicine, Nishinomiya, Japan; 4 Division of Respirology, Chiba Cancer Center, Chiba, Japan; 5 Department of Medical Oncology, Faculty of Medicine, University of Tsukuba, Tsukuba, Japan; 6 Department of Respirology, Graduate School of Medicine, Chiba University, Chiba, Japan; 7 Department of Surgery, School of Medicine, Toho University, Tokyo, Japan; 8 Department of Pathology, Tokyo Women’s Medical University Yachiyo Medical Center, Yachiyo, Japan

**Keywords:** Replication-competent adenovirus, Infectivity, Transcriptional activity, p53 genotype, Biomarker

## Abstract

**Background:**

Replication-competent adenoviruses (Ad) produced cytotoxic effects on infected tumors and have been examined for the clinical applicability. A biomarkers to predict the cytotoxicity is valuable in a clinical setting.

**Methods:**

We constructed type 5 Ad (Ad5) of which the expression of *E1A* gene was activated by a 5′ regulatory sequences of survivin, midkine or cyclooxygenase-2, which were highly expressed in human tumors. We also produced the same replication-competent Ad of which the fiber-knob region was replaced by that of Ad35 (AdF35). The cytotoxicity was examined by a colorimetric assay with human tumor cell lines, 4 kinds of pancreatic, 9 esophageal carcinoma and 5 mesothelioma. Ad infectivity and Ad-mediated gene expression were examined with replication-incompetent Ad5 and AdF35 which expressed the *green fluorescence protein* gene. Expression of cellular receptors for Ad5 and AdF35 was also examined with flow cytometry. A transcriptional activity of the regulatory sequences was investigated with a luciferase assay in the tumor cells. We then investigated a possible correlation between Ad-mediated cytotoxicity and the infectivity/gene expression, the transcriptional activity or the *p53* genotype.

**Results:**

We found that the cytotoxicity was greater with AdF35 than with Ad5 vectors, but was not correlated with the Ad infectivity/gene expression irrespective of the fiber-knob region or the E1A-activating transcriptional activity. In contrast, replication-competent Ad produced greater cytotoxicity in *p53* mutated than in wild-type esophageal carcinoma cells, suggesting a possible association between the cytotoxicity and the *p53* genotype.

**Conclusions:**

Sensitivity to Ad-mediated cytotoxic activity was linked with the p53 genotype but was not lineally correlated with the infectivity/gene expression or the E1A expression.

**Electronic supplementary material:**

The online version of this article (10.1186/s12885-017-3621-x) contains supplementary material, which is available to authorized users.

## Background

A number of clinical trials for cancer therapy with replication-competent viruses have been conducted and some of the agents are approved in China, Unites States and Europe [1, 2]. Adenoviruses (Ad) are one of the agents extensively investigated and are easy to be genetically manipulated to produce replication-restricted types for human tumors. There are mainly 2 structural categories that make preferential replications in tumors, Ad defective of a region that inhibits viral replications in non-tumorous cells such as the E1B 55 kDa-defective type [3] and Ad of which the E1A region is activated with a transcriptional regulatory unit of a gene which is preferentially up-regulated in human tumors. Prediction of Ad-mediated cytotoxicity is important for selecting candidate patients who are suitable for the virotherapy in a clinical setting but such a predictive biomarker for the cytotoxicity remains uncharacterized.

Efficacy of the viral replication-mediated cell death can be influenced by Ad infectivity and also by a transcriptional activity of an exogenous promoter region to activate the E1A region in the second type. Nevertheless, few reports extensively analyzed correlation between the Ad-mediated cytotoxicity and the infectivity or the E1A-activating capacity. On the other hand, further understanding of Ad biology enabled us to produce modified Ad of which the infectivity was changed by replacing the fiber-knob region since the region mediated Ad binding to the cellular receptors [4]. Ad use different receptor molecules, depending on the subtypes. Consequently, substituting the fiber-knob region can convert the infectivity based on the Ad subtypes. Conventional Ad vector belongs to type 5 (Ad5) and uses coxsachie adenovirus receptor (CAR) as the main cellular receptor and intergrin αvβ3 and αvβ5 as the axillar receptor, whereas type 35 Ad (Ad35) vector uses CD46 as the main receptor [5]. Ad5 bearing the Ad35-derived fiber-knob structure (AdF35) therefore infected CD46-positive cells irrespective of CAR expression [6, 7]. The expression levels of CAR molecules in human tumors were variable and often down-regulated, rendering replication-competent Ad5 less cytotoxic to human tumors [8]. In contrast, CD46 was ubiquitously expressed in human cells and the expression was rather up-regulated in a number of human tumors [9]. AdF35 can therefore infect human tumors better than Ad5 [10] and consequently produced greater cytotoxicity [11].

Cytotoxic activities of the replication-competent Ad of which the E1A is regulated by an exogenous regulatory region can also be attributable to transcriptional activities of the region in target cells. We and others previously showed that a 5′ untranslated region of *midkine* (MK) [12], *survivin* (Sur) [13] or *cyclooxygenase-2* (COX-2) gene [14], all of which were up-regulated in the expression in a number of human tumors, activated a reporter gene in human tumors but much less in human normal cells. Replication-competent Ad powered by the promoter region in fact produced preferential cytotoxicity in various type of human tumors with little damages in non-transformed cells [15–17]. Replacement of the fiber-knob region with the Ad35-derived one can widen the target tumor scopes and furthermore produce better cytotoxicity [18]. In a clinical setting, a possible biomarker to predict the efficacy of these Ad is desirable to narrow down candidate patients. We therefore tested the cytotoxicity of replication-competent Ad5 and AdF35 bearing the same transcriptional regulatory region in 3 kinds of human tumors which include 4 pancreatic, 9 esophageal carcinoma and 5 mesothelioma cell lines, and examined whether Ad infectivity and the transactivation activity could be a predictive marker. We also examined a possible linkage between the *p53* genotype and the cytotoxicity with the esophageal carcinoma.

## Methods

### Cells

Human pancreatic carcinoma, PANC-1 (TKG 0606, *p53* genotype: mutated), AsPC-1 (JCRB1454, null), MIA-PaCa-2 (TKG 0227, mutated) and BxPC-3 (JCRB1448, mutated) cells, and human esophageal carcinoma, TE-1 (TGK 0252, mutated at codon 272 Val to Met), TE-2 (TGK 0253, wild-type), TE-10 (TKG 0261, mutated at codon 242 Cys to Tyr), TE-11 (TKG 0262, wild-type), YES-2 (mutated at codon 236 Tyr to Asn) [19], YES-4 (wild-type) [20], YES-5 (mutated at codon 280 Arg to Gly) [20], YES-6 (wild-type) [20] and T.Tn (JCRB 0261, mutated at codon 214 His to Arg and 258 Glu to stop) cells were from Cell Resource Center for Biomedical Research (TKG number; Sendai, Japan), National Institutes of Biomedical Innovation, Health and Nutrition (JCRB number; Tokyo, Japan) or Dr. Yutaka Shimada (YES-2, YES-4, YES-5 and YES-6; Kyoto University, Kyoto, Japan). HEK293 cells (CRL-1573) and human mesothelioma, NCI-H2452 (CRL-5946, wild-type but truncated p53 protein), NCI-H2052 (CRL-5915, wild-type), NCI-H226 (CRL-5826, wild-type), NCI-H28 (CRL-5820, wild-type) and MSTO-211H (CRL-2081, wild-type) cells, were from ATCC (CRL number; Manassas, VA, USA). All the cells were cultured with RPMI 1640 supplemented with 10% fetal calf serum.

### Construction of ad

Replication-incompetent Ad5 expressing the *β green fluorescence protein* gene (GFP) (U55762) powered by cytomegalovirus promoter (Ad5/GFP) were prepared with Adeno-X expression system (Takara, Shiga, Japan), which included ligation of transgene-harboring pShuttle 2 and Adeno-X vectors followed by transfection into HEK293 cells. AdF35, bearing the above transgene (AdF35/GFP or AdF35/LacZ), were produced with the Adeno-X vector of which the corresponding genomic fragment (AY271307 at 30827–33609) was replaced with that of the Ad35 DNA (Avior Therapeutic, Seattle, WA, USA). These replication-incompetent Ad5 and AdF35 vectors used the same cytomegalovirus promoter (BK000394) to activate the respective genes. Replication-competent Ad5 or AdF35 in which the *E1* gene was activated by an exogenous regulatory element, Ad5/Sur, Ad5/MK, Ad5/COX-2, AdF35/Sur, AdF35/MK and AdF35/COX-2, were prepared by replacing the authentic E1 promoter region with 5′ upstream regulatory sequences of the *MK* (0.6 kb, D10604) [12] the *Sur* (0.5 kb, U75285) [13], or *COX-2* (0.3 kb, U04636) gene [14]. Ad were purified with an Adeno-X virus purification kit (BD Biosciences, San Jose, CA, USA) and the numbers of virus particles (vp) per ml was estimated with the formula, absorbance at 260 nm of purified Ad in the presence of 0.1% sodium dodecyl sulfate × 1.1 × 10^12^.

### Cytotoxicity of ad

Cells (5 × 10^3^/well) were seeded in 96-well plates and were cultured for 5 days with different amounts of Ad (vp/cell). Cell viability was determined with a cell proliferation colorimetric WST kit (Wako, Osaka, Japan). The amount of formazan produced was determined with the absorbance at 450 nm and the relative viability was calculated based on the absorbance without any treatments. Half maximal inhibitory concentration (IC_50_) was estimated with CalcuSyn software (Biosoft, Cambridge, UK).

### Ad infectivity/ad-mediated gene expression

Cells were infected with Ad5/GFP or AdF35/GFP at 30 multiplicity of infection (MOI) for 30 min and were washed to remove Ad. Infected cells were cultured for 2 days and then analyzed for percentages of GFP-positive cells with FACSCalibur (BD Biosciences) and CellQuest software (BD Biosciences). Cells of which fluorescence was greater than the brightest 5% of uninfected cells were judged as positively stained.

### Expression of ad receptor molecules

Cells were stained with either anti-CAR antibody (Ab) (#05–644*,* Upstate, Charlottesville, VA, USA) followed by fluorescein isothiocyanate (FITC)-conjugated anti-mouse IgG Ab or with FITC-conjugated anti-human CD46 Ab (#555949, BD Pharmingen, San Jose, CA, USA). They were then analyzed for their fluorescence intensity with FACSCalibur and CellQuest software. Mean fluorescence intensity of the staining profiles was expressed as an arbitrary FL1 unit after standardizing intensity by the second antibody, FITC-conjugated or isotype-matched control Ab as 10 in the unit.

### Transcriptional activity

Genomic fragments of a 5′-transcriptional regulatory region of the 0.6 kb *MK* [12], the 0.5 kb *Sur* [13], or the 0.3 kb *COX-2* [14] gene were cloned into pGL-2 basic vector (Promega, Madison, WI, USA) that contained the *firefly luciferase* gene. Plasmid DNA containing the respective genomic fragments, pGL-control vector (Promega) harboring the SV40 T antigen promoter-linked *firefly luciferase* gene, or pGL-basic vector without any transcriptional regulatory regions (Promega), and a control vector, the *renilla luciferase* gene fused with the *herpes simplex virus-thymidine kinase* gene promoter (pRL-TK, Promega), at a molar ratio of 10:1, was transfected into tumors with a lipofectin reagent (Life Technologies, Gaithersburg, MD, USA). Cell lysate on day 2 was assayed for the luciferase activity with the dual luciferase reporter assay (Promega). The firefly luciferase activity was standardized with the amounts of luminescence produced by renilla luciferase and the relative activity was expressed as a percentage of the SV40 T antigen promoter-mediated activity.

### Western blot analysis

Lysate of cells treated with Ad was subjected to sodium dodecyl sulfate polyacrylamide gel electrophoresis. The protein was transferred to a nylon filter and was hybridized with Ab against γ-H2A histone family member X (γ-H2AX) (#613401, BioLegend, San Diego, CA, USA), p53 (DO-10 MS-187-P, Thermo Fisher Scientific, Fremont, CA, USA), p21 (#2947, Cell Signaling, Danvers, MA, USA) or β-actin (#4970, Cell Signaling) as a control. The membranes were developed with the ECL system (GE Healthcare, Buckinghamshire, UK).

## Results

### Cytotoxicity of replication-competent Ad5 and AdF35

We examined cytotoxic activity of the replication-competent Ad5 and AdF35 on human pancreatic and esophageal carcinoma, and mesothelioma with the WST assay. We compared relative cytotoxicity between Ad5 and AdF35 which were activated by the same transcriptional regulatory region and showed the cytotoxicity with IC_50_ values which were expressed as vp per cell. (Table 1, Additional file 1: Figure S1). The IC_50_ values of AdF35 were in general lower than those of Ad5 irrespective of the regulatory regions. All the 4 pancreatic carcinoma cells showed the sensitivity to replication-competent AdF35 greater than Ad5 driven by MK, Sur and COX-2 regions. In contrast, some of esophageal carcinoma and mesothelioma cells did not produce such sensitivity to AdF35 vectors. Ad5/MK-infected TE-10, TE-11 and YES-4, and Ad5/Sur-infected YES-4 and YES-6 cells in esophageal carcinoma, and Ad5/MK-infected NCI-H2452, NCI-H226 and NCI-H28 cells, Ad5/Sur-infected NCH-226 cells and Ad5/COX-2-infected NCI-H2452 cells in mesothelioma, achieved greater cytotoxicity than corresponding AdF35 vectors. The cases that showed increased sensitive to Ad5 were however relatively limited, only 5 paired cases out of total 27 cases of esophageal carcinoma (9 cell kinds and 3 types of the regulatory regions), and 5 cases out of 15 cases of mesothelioma (5 cell kinds and 3 types). These data collectively showed that AdF35 achieved greater cytotoxic effects than prototype type 5 Ad and suggested that differential infectivity by the fiber-knob replacement influenced the cytotoxicity.

**Table 1:** Cytotoxicity of replication-competent Ad on carcinoma cells

Cells			IC_50_ values (Average ± SE)			
	Ad5/MK	AdF35/MK	Ad5/Sur	AdF35/Sur	Ad5/COX-2	AdF35/COX-2
Pancreatic carcinoma (1 × 10^3^ vp/cell)						
AsPC-1	16.1 ± 2.7	9.2 ± 0.6	15.7 ± 0.5	3.6 ± 0.6	245 ± 41.5	38.8 ± 12.7
PANC-1	36.1 ± 8.8	13.7 ± 6.4	15.9 ± 2.6	2.6 ± 0.2	244 ± 77.1	27.6 ± 0.6
BxPC-3	44.6 ± 3.1	4.1 ± 2.0	64.9 ± 8.8	1.8 ± 0.9	474 ± 9.6	37.1 ± 7.1
MIA-PaCa-2	12.5 ± 2.7	2.2 ± 0.3	105 ± 16.3	8.0 ± 0.9	69.6 ± 5.4	32.1 ± 6.2
Esophageal carcinoma (1 × 10^4^ vp/cell)						
TE-1	32.2 ± 2.4	10.1 ± 2.0	56.0 ± 1.5	9.71 ± 4.7	317 ± 96.5	18.5 ± 1.9
TE-2	244 ± 188	28.6 ± 4.6	100 ± 32.6	15.1 ± 3.9	62.8 ± 19.9	46.6 ± 22.3
TE-10	2.6 ± 1.1	12.3 ± 4.7	27.3 ± 8.6	3.7 ± 0.6	46.1 ± 6.3	8.1 ± 1.6
TE-11	1.5 ± 0.3	3.0 ± 0.5	1.5 ± 0.3	1.1 ± 0.5	30.9 ± 11.8	7.7 ± 1.6
YES-2	5.7 ± 2.4	0.3 ± 0.2	7.6 ± 1.7	1.4 ± 0.6	80.1 ± 18.7	4.0 ± 2.7
YES-4	1.2 ± 0.8	10.6 ± 1.6	2.1 ± 0.3	7.1 ± 2.8	18.8 ± 4.7	5.6 ± 1.0
YES-5	16.7 ± 6.3	1.5 ± 1.0	8.1 ± 3.1	5.8 ± 1.9	37.5 ± 7.8	16.8 ± 5.3
YES-6	424 ± 85.2	157 ± 40.9	89.5 ± 21.5	226 ± 14.5	260 ± 43.1	40 ± 1.9
T.Tn	9.6 ± 0.9	1.0 ± 0.2	36.1 ± 1.2	1.1 ± 0.0	58.2 ± 19.0	6.0 ± 0.7
Mesothelioma (1 × 10^3^ vp/cell)						
NCI-H2452	7.18 ± 1.0	18.2 ± 6.2	43.1 ± 2.9	27.2 ± 9.0	41.6 ± 11.7	50.8 ± 5.8
NCI-H2052	520.6 ± 14.8	143.2 ± 24.8	516.1 ± 11.3	187.2 ± 50.9	>1000	524.0 ± 18.1
NCI-H226	35.1 ± 3.5	59.3 ± 0.6	55.5 ± 1.1	55.7 ± 2.5	550.0 ± 10.0	531.3 ± 11.3
NCI-H28	4.4 ± 0.4	5.6 ± 0.1	45.9 ± 21.1	7.1 ± 0.1	140.3 ± 23.7	36.6 ± 2.1
MSTO-211H	12.6 ± 2.2	0.7 ± 0.0	95.0 ± 19.1	3.9 ± 0.2	450.6 ± 17.2	10.9 ± 2.0

Respective carcinoma cells were infected with Ad at various vp/cell ratios and the cytotoxicity was tested with the WST assay. The experiments were conducted 3 times and the representative data are shown. IC_50_ values were estimated with CalcuSyn software. Averages and SEs are shown (*n* = 3)

### Correlation of ad infectivity and receptor

We used Ad5 and AdF35 vectors expressing the *GFP* gene and tried to show the Ad infectivity with a percentage of GFP-positive cells. The percentages did not directly reflect the Ad infectivity since GFP fluorescence was influenced by the other factors such as the promoter activity to activate the *GFP* gene and GFP protein stability in respective cells. The GFP-positive percentages were therefore indirect estimation of Ad infectivity. We compared the putative infectivity, which included GFP expression ability, between Ad5/GFP and AdF35/GFP in the same cells (Table 2, Additional file 2 Figure S2). The infectivity was greater with AdF35 than with Ad5 vectors in all the tumor cells tested although the enhanced infectivity level by replacing the fiber-knob region was variable among the cells. Infectivity to HEK293 cells was much greater than these tumor cells, and the transduction efficacy with Ad5 and AdF35 was similar at 30 MOI. We noticed that the differential infectivity in NCI-H226 and NCI-H28 cells was also small because these cells were susceptible to Ad5-mediated infection. We then examined an expression level of the major receptor molecules, CAR for Ad5 and CD46 for AdF35 vectors, based on the expression in HEK293 cells as a standard. The CAR expression level of all the tumor cells was lower than that of HEK293 cells, whereas the CD46 level in the tumor cells was greater than that in HEK293 cells except MIA-PaCa-2 and MSTO-211H cells. These data suggested that human tumor cells tested in the present study expressed CD46 relatively well in comparison with CAR molecules and consequently AdF35 infected greater than Ad5 in these tumor cells.

**Table 2:** Infectivity/gene expression of Ad5/GFP and AdF35/GFP, and expression levels of CAR and CD46 on target cells

	Infectivity tested at MOI = 30		Receptor expression	
Cells	Ad5/GFP	AdF35/GFP	CAR	CD46
	(% positive cells^a^)		(% mean fluorescence intensity^b^)	
Pancreatic carcinoma				
HEK293	98.98 ± 0.11	99.47 ± 0.11	100	100
AsPC-1	8.47 ± 0.27	25.32 ± 0.57	71.6	182.4
PANC-1	7.66 ± 0.50	28.35 ± 0.41	65.5	175.7
BxPC-3	19.48 ± 0.62	68.95 ± 0.45	26.8	231.1
MIA-PaCa-2	5.89 ± 0.54	65.41 ± 0.68	2.0	62.6
Esophageal carcinoma				
HEK293	87.20±0.52	78.05±0.70	100	100
TE-1	8.06±1.17	53.47±0.10	11.5	321.5
TE-2	0.79±0.14	10.15±0.44	33.7	160.2
TE-10	16.15±0.52	35.33±0.67	25.9	99.7
TE-11	22.86±0.53	42.82±0.74	39.1	290.2
YES-2	5.09±1.29	51.54±0.36	0.3	131.7
YES-4	27.18±0.16	61.23±0.07	47.2	135.2
YES-5	22.18±0.32	69.97±0.89	27.0	176.3
YES-6	16.59±0.25	27.63±0.17	53.1	120.4
T.Tn	0.49±1.00	21.60±0.15	14.3	208.9
Mesothelioma				
HEK293	93.7±0.1	93.8±0.4	100	100
NCI-H2452	49.8±0.3	75.3±0.6	35.6	117.6
NCI-H2052	7.4±0.3	81.5±0.5	0.7	174.7
NCI-H226	78.5±1.2	81.9±1.5	84.0	164.7
NCI-H28	85.5±0.4	95.1±0.2	17.8	150.4
MSTO-211H	8.7±0.3	67.9±0.9	2.3	56.0

Cells infected with Ad5/GFP or AdF35/GFP at 30 MOI were analyzed for the fluorescence intensity with flow cytometry. ^a^Positively stained cells were defined as those that showed fluorescence greater than the brightest 5% of uninfected cells. Averages and the SEs are shown (*n* = 3). ^b^CAR and CD46 expression levels were determined with flow cytometry and are expressed with arbitrary unit. The intensity is expressed as a percentage of that of HEK293 cells. Three tumor types were respectively examined with HEK293 cells as a control

We then investigated any possible correlation between Ad infectivity/gene expression and the receptor expression level (Table 3, Additional file 3: Figure S3 for individual data). The correlation coefficient between CAR levels and Ad5/GFP was −0.13 (*P* = 0.87) in pancreatic carcinoma, 0.62 (*P* = 0.07) in esophageal carcinoma and 0.68 (*P* = 0.21) in mesothelioma, indicating no significant correlation in all the cells tested. The correlation coefficient between CD46 and AdF35/GFP was also not significant, −0.20 (*P* = 0.80) in pancreatic carcinoma, 0.14 (*P* = 0.71) in esophageal carcinoma and 0.72 (*P* = 0.16) in mesothelioma. These data clearly showed no linear correlation between the major receptor expression level and the Ad infectivity/gene expression in both Ad5 and AdF35 vectors.

**Table 3:** Correlation between Ad infectivity/gene expression and receptor expression

Tumor type	Infectivity	Receptor expression	Correlation	*P* value
	(% positive cells)	(% mean fluorescence intensity)	coefficient	
Pancreatic carcinoma				
	Ad5/GFP	CAR	−0.13	0.87
	AdF35/GFP	CD46	−0.20	0.80
Esophageal carcinoma				
	Ad5/GFP	CAR	0.62	0.07
	AdF35/GFP	CD46	0.14	0.71
Mesothelioma				
	Ad5/GFP	CAR	0.68	0.21
	AdF35/GFP	CD46	0.72	0.16

Infectivity data and receptor expression levels of respective tumor types (pancreas; 4 cells, esophagus; 9 cells, mesothelioma; 5 cells) are derived from Table 2

### Correlation of ad infectivity and ad-mediated cytotoxicity

We next investigated possible effects of Ad infectivity/gene expression on the cytotoxicity produced by Ad with different transcriptional regulatory elements. We used average IC_50_ values as the cytotoxicity by respective replication-competent Ad and GFP-positive percentages as the infectivity, and calculated correlation coefficients among 4 pancreatic, 9 esophageal and 5mesothelioma cells (Table 4, Additional file 4: Figure S4 for individual data). Analyses with pancreatic carcinoma cells showed positive correlation except AdF35/MK-mediated cytotoxicity but none of them were statistically significant, and those with all the esophageal carcinoma had negative correlation without statistical significance. A half of mesothelioma cases was positively and the other was negatively correlated, and none of them was statistical significant. These data clearly indicated no significant linear correlation between the cytotoxicity and the Ad infectivity/gene expression.

**Table 4:** Correlation between Ad-mediated cytotoxicity and Ad infectivity/gene expression

Tumor type	Infectivity^b^	Correlation coefficient	*P* value
Ad-mediated cytotoxicity^a^			
Pancreatic carcinoma			
Ad5/MK	Ad5/GFP	0.77	0.22
Ad5/Sur	Ad5/GFP	0.05	0.95
Ad5/COX-2	Ad5/GFP	0.94	0.06
AdF35/MK	AdF35/GFP	−0.89	0.11
AdF35/Sur	AdF35/GFP	0.31	0.69
AdF35/COX-2	AdF35/GFP	0.14	0.86
Esophageal carcinoma			
Ad5/MK	Ad5/GFP	−0.15	0.70
Ad5/Sur	Ad5/GFP	−0.52	0.15
Ad5/COX-2	Ad5/GFP	−0.22	0.56
AdF35/MK	AdF35/GFP	−0.36	0.34
AdF35/Sur	AdF35/GFP	−0.28	0.47
AdF35/COX-2	AdF35/GFP	−0.53	0.13
Mesothelioma			
Ad5/MK	Ad5/GFP	−0.57	0.31
Ad5/Sur	Ad5/GFP	−0.65	0.24
Ad5/COX-2	Ad5/GFP	−0.18	0.82
AdF35/MK	AdF35/GFP	0.10	0.87
AdF35/Sur	AdF35/GFP	0.07	0.91
AdF35/COX-2	AdF35/GFP	0.15	0.82

^a^Cytotoxicity data are expressed as IC_50_ values of respective cells, 4 pancreatic, 9 esophageal carcinoma and 5 mesothelioma cells as shown in Table 1

^b^Infectivity data are percentages of GFP-positive cells infected at Ad5/GFP or AdF35/GFP at 30 MOI as shown in Table 2

### Correlation of transcriptional activity for E1 activation and ad-mediated cytotoxicity

We further examined a transcriptional activity of the regulatory regions which were used for activation of E1 region genes in target tumor cells (Table 5, Additional file 5: Figure S5). We tested a luciferase activity powered by the same promoter region in respective cells. The activity did not precisely reflect the E1-activating ability in replicating Ad since Ad proteins produced during the viral replications influenced the ability. We therefore used the luciferase activity as a putative E1 transcription marker in the present study. The transcriptional activity was expressed as a percentage of that of SV40 T antigen and the relative activity showed that MK- and COX-2-mediated activities were greater than SV40 T antigen-mediated activity irrespective of cell types. In contrast, Sur-mediated activities were much variable depending on cells tested.

**Table 5:** Transcriptional activity of the regulatory region in target cells

	Transcriptional activity (% activity of SV40 T antigen)		
Cells	MK	Sur	COX-2
Pancreatic carcinoma			
AsPC-1	345 ± 59.9	75.6 ± 2.9	212 ± 11.5
PANC-1	224 ± 3.6	69.8 ± 4.28	243 ± 11.4
BxPC-3	454 ± 49.1	67.8 ± 10.6	363 ± 86.5
MIA-PaCa-2	182 ± 22.4	135 ± 7.0	783 ± 27.6
Esophageal carcinoma			
TE-1	400 ± 99.0	162 ± 13.6	752 ± 71.9
TE-2	375 ± 7.9	610 ± 136.5	625 ± 28.1
TE-10	339 ± 26.9	906 ± 34.9	431 ± 59.5
TE-11	702 ± 30.2	6770 ± 526.0	410 ± 128.0
YES-2	314 ± 20.2	1790 ± 156.0	351 ± 27.1
YES-4	187 ± 2.6	310 ± 13.2	282 ± 18.5
YES-5	745 ± 47.7	395 ± 11.2	160 ± 12.5
YES-6	540 ± 7.4	3110 ± 96.8	417 ± 31.4
T.Tn	199 ± 32.0	2410 ± 233.0	126 ± 10.2
Mesothelioma			
NCI-H2452	153 ± 45.7	35.0 ± 5.0	291 ± 72.4
NCI-H2052	189 ± 4.5	38.6 ± 1.0	233 ± 10.1
NCI-H226	112 ± 4.2	7.2 ± 0.3	381 ± 61.2
NCI-H28	348 ± 68.2	173 ± 62.1	250 ± 38.2
MSTO-211H	168 ± 18.4	134 ± 7.3	141 ± 14.0

Cells were transfected with plasmid vector DNA containing a regulatory region linked with the *luciferase* gene and the transcriptional activity was expressed as a percent luciferase activity of the SV40 T antigen. Three histological types were respectively examined with SV40 T antigen as a control. Averages and SEs are shown (*n* = 3)

We then investigated correlation between the cytotoxicity and the E1-activating ability (Table 6, Additional file 6: Figure S6 for individual data). We used average IC_50_ values as the Ad-mediated cytotoxicity and an average luciferase activity as a putative transcriptional activity. Analyses of pancreatic carcinoma showed that correlation coefficient was variable and only one case, cytotoxicity with AdF35/Sur and the Sur-mediated luciferase activity, was statistically correlated. Esophageal carcinoma and mesothelioma cells showed also variability between the cytotoxicity and the transcriptional activity, and none of them was statistically correlated. These data collectively demonstrated that the cytotoxicity was in general independent of the transcriptional activity of the regulatory regions.

**Table 6:** Correlation between Ad-mediated cytotoxicity and the transcriptional activity used in E1 activation

Tumor type	Transcriptional	Correlation coefficient	*P* value
Ad-mediated cytotoxicity^a^	activity^b^		
Pancreatic carcinoma			
Ad5/MK	MK	0.57	0.43
Ad5/Sur	Sur	0.80	0.20
Ad5/COX-2	COX-2	−0.58	0.42
AdF35/MK	MK	−0.16	0.84
AdF35/Sur	Sur	0.99	0.01^c^
AdF35/COX-2	COX-2	−0.18	0.81
Esophageal carcinoma			
Ad5/MK	MK	0.17	0.67
Ad5/Sur	Sur	−0.18	0.65
Ad5/COX-2	COX-2	0.60	0.09
AdF35/MK	MK	0.18	0.65
AdF35/Sur	Sur	0.19	0.62
AdF35/COX-2	COX-2	0.48	0.19
Mesothelioma			
Ad5/MK	MK	−0.07	0.92
AdF35/Sur	Sur	−0.27	0.66
Ad5/COX-2	COX-2	0.08	0.92
AdF35/MK	MK	−0.25	0.68
Ad5/Sur	Sur	−0.53	0.36
AdF35/COX-2	COX-2	0.54	0.35

^a^Cytotoxicity data are expressed as IC_50_ values of respective cells, 4 pancreatic, 9 esophageal and 5 mesothelioma cell lines as shown in Table 1

^b^Transcriptional activity data are percentages of the SV40 T antigen promoter-mediated luciferase activity as shown in Table 5

^c^Statistically significant

### Correlation of *p53* genotype and ad-mediated cytotoxicity

**Fig. 1:**
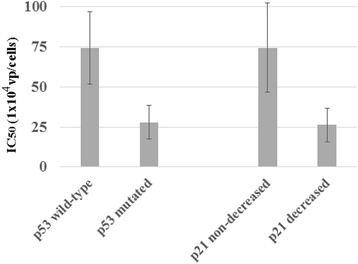
Cytotoxicity of esophageal carcinoma to replication-competent Ad in terms of the *p53* genotypes and p21 responses to cisplatin treatments. Ad-mediated cytotoxicity was expressed as IC_50_ values, which was tested with Ad5/MK, AdF35/MK, Ad5/Sur, AdF35/Sur, Ad5/COX-2 and AdF35/COX-2. A response of p21 levels to cisplatin was judged from Western blot analysis in Fig. 2. Non-decreased cells were YES-2, YES-4 and YES-6, and decreased cells were TE-1, TE-10, TE-11, YES-5 and T.Tn cells

We therefore examined a possible linkage between the *p53* genotype and the cytotoxicity. The p53 genotype of all the pancreatic carcinoma was either mutated (PANC-1, BxPC-3 and MIA-PaCa-2) or deleted (AsPC-1), and that of all the mesothelioma was wild-type except NCI-H2452 cells which had truncated p53 protein despite the wild-type *p53* gene. The distribution of the *p53* genotypes in pancreatic carcinoma and mesothelioma was not even, which made it difficult to analyze the linkage in the same tumor type. We then investigated cytotoxicity of esophageal carcinoma, which included 5 cells with mutated (TE-1, TE-10, YES-2, YES-5 and T.Tn) and 4 cells with wild-type *p53* gene (TE-2, TE-11, YES-4 and YES-6). We examined IC_50_ values produced by all the replication-competent Ad irrespective of the regulatory regions with regard to the *p53* genotype. The IC_50_ values tested with *p53*-wild-type cells (average ± SE: 74.4 ± 22.5 × 10^4^ vp/cell) (*n* = 24; 4 cells × 2 vectors with different fiber-knob region × 3 regulatory regions) were greater than those of *p53*-mutated cells (28.0 ± 10.6, *n* = 30) (*P* = 0.05) (Fig. 1). AdF35 vectors showed more statistical difference (*P* = 0.04) with greater IC_50_ values in the wild-type (45.7 ± 20.6, *n* = 12) than in mutant *p53* gene (6.7 ± 1.5, *n* = 15). In contrast, Ad5 vectors did not show the statistical difference (*P* = 0.21) with those in the wild-type (103.0 ± 39.2, *n* = 12) and in mutant p53 gene (49.4 ± 20.0, *n* = 15), but Ad5 tended to be more effective to *p53*-mutant than the wild-type cells.

**Fig. 2:**
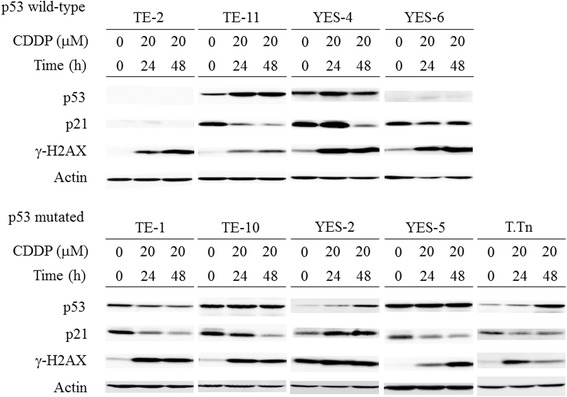
Western blot analysis of esophageal carcinoma treated with cisplatin. Cells were treated with 20 μM cisplatin for 24 or 48 h and the lysate was subjected to gel electrophoresis. Expression of molecules was probed with respective Ab and actin was used as a loading control

We further investigated whether p21 might play a role in the cytotoxicity since previous studies showed controversial data regarding the correlation [21, 22]. We treated cells with cisplatin, a DNA damaging agent, and examined a change of p21 expression (Fig. 2). Cisplatin induced DNA damages, which was evidenced by increased γ-HX2A expression. Cells with wild-type *p53* gene in general increase the p53 expression but only TE-11 and YES-4 cells augmented the expression (Additional file 7 Figure S7). Cells with mutated *p53* gene showed various responses, decreased in TE-1, increased in YES-2 and T.Tn, and unchanged p53 in TE-10 cells. The differential p53 responses were probably attributable to varied ubiquitination levels of p53 or distinct p53 upstream pathways which mediate p53 phosphorylation in respective cells. A change of p21 expression was different from that of p53 although p21 is one of the p53 targets. The p21 levels decreased in TE-1, TE-10, TE-11, YES-5 and T.Tn cells, but increased in YES-2, even if temporally in YES-4 (Additional file 7 Figure S7). YES-6 cells remained unchanged and TE-2 cells were undetectable for p21. We tentatively classified the cells into a p21-decreased group or non-decreased group (YES-2, YES-4 and YES-6), and examined any correlation between the p21 expression change and the cytotoxicity. The IC_50_ values in the p21-decreased group (26.3 ± 10.5, *n* = 30) were marginally different those in the p21 non-decreased group (74.5 ± 28.0, *n* = 18) (*P* = 0.06) (Fig. 1). AdF35 vectors showed statistical difference (*P* = 0.05) with greater IC_50_ values in the non-decreased group (50.2 ± 27.6, *n* = 9) than in the decreased group (7.1 ± 1.4, *n* = 15). In contrast, Ad5 vectors did not show the statistical difference (*P* = 0.26) with those in the non-decreased group (98.8 ± 49.2, *n* = 9) and in the decreased group (45.4 ± 25.4, *n* = 15). These data collectively suggested that cells with wild-type *p53* genotype were more resistant to replication-competent Ad than those with mutated *p53* genotype, and cells with decreased p21 levels responding to DNA damages were more sensitive than non-decreased cells to the cytotoxicity.

## Discussion

We investigated in the present study a possible correlation between cytotoxicity produced by replication-competent Ad and the infectivity/gene expression or a transcriptional activity of an exogenous regulatory region that was used to activate E1 region genes. We produced AdF35 which differed only in the fiber-knob region and compared with the prototype Ad5 in the cytotoxicity and the infectivity. The present study demonstrated that replication-competent AdF35 produced greater cytotoxicity than the prototype Ad5 bearing the same regulatory region, but the cytotoxicity irrespective of the Ad types was not correlated with the infectivity/gene expression or transcriptional activity of the region used for activation of E1 genes. Nevertheless, we demonstrated that the *p53* genotype differentiated the sensitivity of esophageal carcinoma to the Ad-mediated cytotoxicity with greater cytotoxicity in *p53*-mutated cells than in *p53* wild-type cells.

A biomarker to predict an oncolytic ability of replication-competent Ad is important in the clinical applications. The biomarker is useful to select a patient who responds to the Ad-mediate cancer therapy and to exclude a patient who suffers from severe adverse effects caused by the gene medicine. Improved cytotoxicity of AdF35 in comparison with the corresponding Ad5 could be attributable to the enhanced infectivity. Expression of CAR molecules, the major cellular receptor of Ad5, was often down-regulated in human tumors and in fact the present study showed that the CAR expression levels in 3 kinds of human tumors were lower than that of HEK293 cells. In contrast, the level of CD46 molecules, the major receptor for Ad35, did not decrease in the tumors and was rather higher than that in HEK293 cells. The fiber-knob region of Ad5 and Ad35 is responsible for binding with CAR and CD46 molecules, respectively, and replacement of the Ad5-derived region with the Ad35 region ablated the CAR binding ability and enabled AdF35 bind to CD46 molecules. Nevertheless, cytotoxicity of replication-competent Ad5 or AdF35 was not directly correlated with infectivity/gene expression of Ad5 or AdF35 vector irrespective of the transcriptional regions used. These data suggest that expression of subsidiary Ad receptors such as integrin αvβ3 and αvβ5 can also be pivotal for Ad infectivity/gene expression. The present study demonstrated that GFP-positive percentages produced by Ad5 or AdF35 vector were unrelated with CAR and CD46 levels, respectively. Ad-mediated gene expression is regulated at various steps and the infection process can also be influenced by a threshold of the receptor expression. The GFP expression was thereby not directly or linearly associated with the major receptor expression levels. Contribution of the major receptors to the infectivity can be limited in particular in cells with CAR-low or CD46-low expression. Lyle et al. in fact demonstrated that integrin αvβ5 worked as the primary receptor in CAR-negative cells [23]. Previous studies also suggested that infectivity of Ad5 or Ad5 bearing type 11-derived fiber-knob region, which used CD46 molecules as the major receptor, was not directly correlated with the cytotoxicity although the studies did not analyzed statistically [24]. Increased CAR expression augmented Ad5 infectivity and the Ad5-mediated cytotoxicity [25, 26], but correlation between the CAR expression and Ad5 infectivity was not extensively investigated. In contrast, the present study statistically demonstrated that increased Ad infectivity/gene expression was not associated with the Ad replication-mediated cytotoxicity.

We also analyzed the E1-activating ability of Ad in the infected cells since E1A protein or the transcript levels were linked with the cytotoxic activity of the Ad [24, 27]. The present study showed that the transcriptional activity of respective regulatory regions varied depending on target cells and the region integrated in Ad. The activities of MK and COX-2 were constantly greater than that of SV40 T antigen, whereas the Sur activity was variable in comparison with that of SV40 T antigen. The variability of Sur activities in the tumors tested could be partly attributable to preferential expression of the *Sur* gene at G2/M phase [28]. Nevertheless, the present study demonstrated that the E1A-activation ability was not directly correlated with the cytotoxicity except one case, AdF35/Sur-mediated cytotoxicity and Sur activity in pancreatic carcinoma. The previous studies which analyzed a possible linkage between E1A expression and the cytotoxicity did not analyze statistical significance [24, 27], but the present study was to our knowledge the first report to demonstrate no significant association between them. These data consequently suggest that a cellular factor play an important role in the Ad-mediated cytotoxicity. A mechanism of Ad replication-induced cell death is complex and the cell death pathways might be different among the cells tested.

We examined a correlation between the *p53* genotype and the cytotoxic activity with esophageal carcinoma and demonstrated that cells with wild-type *p53* gene were resistant to Ad replication-induced cytotoxicity compared with those with mutant *p53* in particular with the AdF35 vectors. Previous studies showed that transduction with the wild-type *p53* enhanced cytotoxicity produced by replication-competent Ad [29, 30] and that the Ad-mediated cytotoxicity, which was further augmented by co-expressed p53, was independent of the p53 status of target cells [30, 31]. Expression of E1A accompanied by the viral replications enhanced expression of p53 and the phosphorylation, which contributed to augmentation of cell death. In contrast, the E1A-induced phosphorylation of mutated p53, functioned as a dominant-negative form, increased the resistance to cell death, which consequently augmented viral replications and production of viral progenies. The differential susceptibility of replication-competent Ad in terms of the *p53* genotype can be attributable to how infected cells were subjected to death and to how much viral progenies were produced through preventing premature cell death. A number of factors were involved in a balance between survival and death signals, such as differential activities between apoptotic and anti-apoptotic pathways, and autophagy and anti-autophagy pathways, as well as cellular components that influence viral progeny production. Further investigations, for example, a treatment with siRNA for p53, are required to clarify a possible role of p53 in the Ad-mediated cytotoxicity.

Functional significance of p21, one of the p53 down-stream molecules, in the Ad-mediated cytotoxicity was controversial. Flak et al. showed that cells with inducible p21 were susceptible to replication-competent Ad [21], whereas Höri et al. demonstrated that a chemical agent to augment p21 expression decreased the cytotoxicity [22]. We treated cells with cisplatin, a representative agent to induce DNA damages, and examined p21 expression. Cells infected with replication-competent Ad were not used since they were difficult to be standardized for the DNA damages during viral replications. Increased p21 expression can inhibit cell cycle progression at G1 phase and consequently decreases viral replications. On the other hand, p21 is inhibitory to cell death, which makes cells alive and productive of viral particles. A functional role of p21 in viral replications and the cytotoxicity is thus divalent and can be differentially influenced by properties of the infected cells. The current study showed that decreased p21 expression after DNA damages was associated with increased susceptibility to Ad-mediated cytotoxicity and indicated that down-regulation of p21 facilitated cell cycle progression to make cells competent for viral replications and favored cell death. Biological significance of the down-regulated p21 in terms of the cytotoxicity needs further studies since Ad-mediated cytotoxicity is influenced not only by viral replications but susceptibility to cell death mechanisms. We noticed that the mutated *p53* esophageal carcinoma tended to decreased p21 expression, and consequently mutated *p53* genotype and decreased p21 levels could be relevant to each other regarding the Ad-mediated cytotoxicity. In addition, responses of p53 and p21 to cisplatin in esophageal carcinoma were different from typical damage responses, which suggested that the p53 pathways were impaired. We however found that cisplatin-treated cells induced cleavage of PARP and caspase-3 in all the *p53* wild-type esophageal carcinoma (Additional file 8: Figure S8), indicating that apoptosis was induced by cisplatin. We also showed that association of the cytotoxicity with the *p53* genotype or with the p21 responses was greater with AdF35 than with Ad5 vectors, but the mechanism behind this vector difference was currently unknown.

The present study suggested the *p53* genotype as a potential biomarker to predict the efficacy but this outcome needs to be confirmed with clinical specimens. Moreover, expression of cellular proteins necessary for Ad replications such as nuclear factor-1 and production of type I interferon followed by Ad infection are also issues to be examined since these factors also influence susceptibility of target cells to Ad-mediated cell death [32–34]. Prediction of Ad-mediated cytotoxicity is important from the standpoint of the possible clinical application, and further investigations are required to establish such predictive markers because genetic and epigenetic alterations in target cells are involved in the Ad-mediated cytotoxicity.

## Conclusions

We examined biomarkers that could influence Ad-mediated cytotoxicity. We initially presumed that Ad infectivity/gene expression and transcriptional activity of the regulatory regions played a certain role in the cytotoxicity, but the present analyses showed that these factors were scarcely correlated with the cytotoxicity. We however demonstrated that the cytotoxicity was greater in *p53* mutated than in wild-type esophageal carcinoma cells and perhaps was associated decreased p21 levels.

## Additional file

Additional file 1: Figure. S1. Cytotoxicity of replication-competent Ad on carcinoma cells. The same data in Table 1 were used. Averages and SEs are shown (*n* = 3) (PDF 128 kb)
Additional file 2: Figure. S2. Infectivity/gene expression of Ad5/GFP and AdF35/GFP. Cells infected with Ad5/GFP or AdF35/GFP at 30 MOI were analyzed for the fluorescence intensity with flow cytometry. The same data in Table 2 were used. Averages and SEs are shown (*n* = 3) (PDF 121 kb)
Additional file 3: Figure S3. Individual data of CAR or CD46 expression levels and percent GFP-positive cells in respective cells. The summary of correlation coefficient is shown in Table 3. Correlation coefficient (C) and *P* value are also shown (PDF 79 kb)
Additional file 4: Figure S4. Individual data of Ad-mediated cytotoxicity and percent GFP-positive cells in respective cells. The summary of correlation coefficient is shown in Table 4. Correlation coefficient (C) and *P* value are also shown (PDF 173 kb)
Additional file 5: Figure S5. Transcriptional activity of the regulatory region in target cells. The same data in Table 5 were used. Averages and SEs are shown (*n* = 3) (PDF 155 kb)
Additional file 6: Figure S6. Individual data of Ad-mediated cytotoxicity and transcriptional activity in respective cells. The summary of correlation coefficient is shown in Table 6. Correlation coefficient (C) and *P* value are also shown (PDF 177 kb)
Additional file 7: Figure S7. Quantification of p53 and p21 expression of Fig. 2. Intensity of respective bands were measured with an image analyzer software, ImageJ (https://imagej.nih.gov/ij/). Relative intensity of p53 and p21 was adjusted by actin intensity and shown as a percentage of cisplatin (CDDP)-untreated cells. Expression of p53 and p21 in TE-2 cells was almost undetectable and the intensity data were not included (PDF 131 kb)
Additional file 8: Figure S8. Cisplatin (CDDP) induced cleavages of caspase-3 and PARP. Esophageal carcinoma cells with the wild-type *p53* genotype were treated with CDDP as shown and the cell lysate was probed with the antibody as indicated, poly (ADP-ribose) polymerase (PARP) (can also detect cleaved PARP, #9542), and cleaved caspase-3 (can also detect caspase-3, #9661) (Cell Signaling). Actin was used as a loading control and the blot was the same as that in Fig. 2. Data of untreated YES-6 cells were taken in the same blot as other data (YES-6 cells treated with CDDP), but the sample was loaded next to that of YES-6 cells treated with CDDP for 48 h. The data untreated YES-6 cells were therefore moved to before the data of YEST-6 cells treated with CDDP for 24 h (PDF 118 kb)

## Abbreviations

Ab: Antibody
Ad: Adenoviruses
Ad35: Adenovirus type 35
Ad5: Adenovirus type 5
Ad5/GFP, AdF35/GFP: Ad5 or AdF35 expressing *green fluorescence protein* gene
AdF35: Adenovirus vector bearing the type-35-derived fiber-knob structure
CAR: Coxsachie adenovirus receptor
COX-2: Cyclooxygenase-2
FITC: Fluorescein isothiocyanate
IC_50_: Half maximal inhibitory concentration
MK: Midkine
MOI: Multiplicity of infection
Sur: Survivin
Vp: Virus particles
γ-H2AX: γ-H2A histone family member X

## References

[1] Ma G, Shimada H, Hiroshima K, Tada Y, Suzuki N, Tagawa M (2009). Gene medicine for cancer treatment: commercially available medicine and accumulated clinical data in China. Drug Des Dev Ther.

[2] Ott PA, Hodi FS (2016). Talimogene laherparepvec for the treatment of advanced melanoma. Clin Cancer Res.

[3] Bischoff JR, Kirn DH, Williams A, Heise C, Horn S, Muna M, Ng L, Nye JA, Sampson-Johannes A, Fattaey A, McCormick F (1996). An adenovirus mutant that replicates selectively in p53-deficient human tumor cells. Science.

[4] Shayakhmetov DM, Lieber A (2000). Dependence of adenovirus infectivity on length of the fiber shaft domain. J Virol.

[5] Sharma A, Li X, Bangari DS, Mittal SK (2009). Adenovirus receptors and their implications in gene delivery. Virus Res.

[6] Ni S, Gaggar A, Di Paolo N, Li ZY, Liu Y, Strauss R, Sova P, Morihara J, Feng Q, Kiviat N, Touré P, Sow PS, Lieber A (2006). Evaluation of adenovirus vectors containing serotype 35 fibers for tumor targeting. Cancer Gene Ther.

[7] Kim SY, Kang S, Song JJ, Kim JH (2013). The effectiveness of the oncolytic activity induced by Ad5/F35 adenoviral vector is dependent on the cumulative cellular conditions of survival and autophagy. Int J Oncol.

[8] Pong RC, Lai YJ, Chen H, Okegawa T, Frenkel E, Sagalowsky A, Hsieh JT (2003). Epigenetic regulation of coxsackie and adenovirus receptor (CAR) gene promoter in urogenital cancer cells. Cancer Res.

[9] Anderson BD, Nakamura T, Russell SJ, Peng KW (2004). High CD46 receptor density determines preferential killing of tumor cells by oncolytic measles virus. Cancer Res.

[10] Yu L, Takenobu H, Shimozato O, Kawamura K, Nimura Y, Seki N, Uzawa K, Tanzawa H, Shimada H, Ochiai T, Tagawa M (2005). Increased infectivity of adenovirus type 5 bearing type 11 or type 35 fibers to human esophageal and oral carcinoma cells. Oncol Rep.

[11] Suzuki T, Kawamura K, Li Q, Okamoto S, Tada Y, Tatsumi K, Shimada H, Hiroshima K, Yamaguchi N, Tagawa M (2014). Mesenchymal stem cells are efficiently transduced with adenoviruses bearing type 35-derived fibers and the transduced cells with the IL-28A gene produces cytotoxicity to lung carcinoma cells co-cultured. BMC Cancer.

[12] Miyauchi M, Yoshida Y, Tada Y, Narita M, Maeda T, Bahar R, Kadomatsu K, Muramatsu T, Matsubara S, Nakagawara A, Sakiyama S, Tagawa M (2001). Expression of herpes simplex virus-thymidine kinase gene controlled by a promoter region of the midkine gene confers selective cytotoxicity to ganciclovir in human carcinoma cells. Int J Cancer.

[13] Kawamura K, Yu L, Tomizawa M, Shimozato O, Ma G, Li Q, Sato A, Yang Y, Suzuki T, Abdel-Aziz NM, Tagawa M (2007). Transcriptional regulatory regions of the survivin gene activate an exogenous suicide gene in human tumors and enhance the sensitivity to a prodrug. Anticancer Res.

[14] Yamamoto M, Alemany R, Adachi Y, Grizzle WE, Curiel DT (2001). Characterization of the cyclooxygenase-2 promoter in an adenoviral vector and its application for the mitigation of toxicity in suicide gene therapy of gastrointestinal cancers. Mol Ther.

[15] Yu L, Hamada K, Namba M, Kadomatsu K, Muramatsu T, Matsubara S, Tagawa M (2004). Midkine promoter-driven suicide gene expression and -mediated adenovirus replication produced cytotoxic effects to immortalized and tumour cells. Eur J Cancer.

[16] Ono HA, Davydova JG, Adachi Y, Takayama K, Barker SD, Reynolds PN, Krasnykh VN, Kunisaki C, Shimada H, Curiel DT, Yamamoto M (2005). Promoter-controlled infectivity-enhanced conditionally replicative adenoviral vectors for the treatment of gastric cancer. J Gastroenterol.

[17] Van Houdt WJ, Haviv YS, Lu B, Wang M, Rivera AA, Ulasov IV, Lamfers ML, Rein D, Lesniak MS, Siegal GP, Dirven CM, Curiel DT, Zhu ZB (2006). The human survivin promoter: a novel transcriptional targeting strategy for treatment of glioma. J Neurosurg.

[18] Takagi-Kimura M, Yamano T, Tamamoto A, Okamura N, Okamura H, Hashimoto-Tamaoki T, Tagawa M, Kasahara N, Kubo S (2013). Enhanced antitumor efficacy of fiber-modified, midkine promoter-regulated oncolytic adenovirus in human malignant mesothelioma. Cancer Sci.

[19] Nakamura M, Murakami T, Sakata K, Kusanagi H, Saeki T, Uchisako H, Hayashi H, Tangoku A, Suzuki T (1994). Establishment and characterization of a new human esophageal cancer cell line (YES-2). Bull Yamaguchi Med Sch.

[20] Oka M, Hirose K, Iizuka N, Aoyagi K, Yamamoto K, Abe T, Hazama S, Suzuki T (1995). Cytokine mRNA expression patterns in human esophageal cancer cell lines. J Interf Cytokine Res.

[21] Flak MB, Connell CM, Chelala C, Archibald K, Salako MA, Pirlo KJ, Lockley M, Wheatley SP, Balkwill FR, McNeish IA (2010). p21 promotes oncolytic adenoviral activity in ovarian cancer and is a potential biomarker. Mol Cancer.

[22] Höti N, Chowdhury W, Hsieh JT, Sachs MD, Lupold SE, Rodriguez R (2006). Valproic acid, a histone deacetylase inhibitor, is an antagonist for oncolytic adenoviral gene therapy. Mol Ther.

[23] Lyle C, McCormick F (2010). Integrin αvβ5 is a primary receptor for adenovirus in CAR-negative cells. Virol J.

[24] Wong HH, Jiang G, Gangeswaran R, Wang P, Wang J, Yuan M, Wang H, Bhakta V, Müller H, Lemoine NR, Wang Y (2012). Modification of the early gene enhancer promoter improves the oncolytic potency of adenovirus 11. Mol Ther.

[25] Kang S, Kim JH, Kim SY, Kang D, Je S, Song JJ (2014). Establishment of a mouse melanoma model system for the efficient infection and replication of human adenovirus type 5-based oncolytic virus. Biochem Biophys Res Commun.

[26] Ingemarsdotter CK, Tookman LA, Browne A, Pirlo K, Cutts R, Chelela C, Khurrum KF, Leung EY, Dowson S, Webber L, Khan I, Ennis D, Syed N, Crook TR, Brenton JD, Lockley M, McNeish IA (2015). Paclitaxel resistance increases oncolytic adenovirus efficacy via upregulated CAR expression and dysfunctional cell cycle control. Mol Oncol.

[27] Wang Y, Thorne S, Hannock J, Francis J, Au T, Reid T, Lemoine N, Kirn D, Halldén G (2005). A novel assay to assess primary human cancer infectibility by replication-selective oncolytic adenoviruses. Clin Cancer Res.

[28] Ling X, Bernacki RJ, Brattain MG, Li F (2004). Induction of survivin expression by taxol (paclitaxel) is an early event, which is independent of taxol-mediated G2/M arrest. J Biol Chem.

[29] Chen W, Wu Y, Liu W, Wang G, Wang X, Yang Y, Chen W, Tai Y, Lu M, Qian Q, Zhang Q, Chen G (2011). Enhanced antitumor efficacy of a novel fiber chimeric oncolytic adenovirus expressing p53 on hepatocellular carcinoma. Cancer Lett.

[30] Mitlianga PG, Sioka C, Vartholomatos G, Goussia A, Polyzoidis K, Rao JS, Kyritsis AP (2006). p53 enhances the Delta-24 conditionally replicative adenovirus anti-glioma effect. Oncol Rep.

[31] Ohashi M, Kanai F, Tateishi K, Taniguchi H, Marignani PA, Yoshida Y, Shiratori Y, Hamada H, Omata M (2001). Target gene therapy for alpha-fetoprotein-producing hepatocellular carcinoma by E1B55k-attenuated adenovirus. Biochem Biophys Res Commun.

[32] de Jong RN, van der Vliet PC (1999). Mechanism of DNA replication in eukaryotic cells: cellular host factors stimulating adenovirus DNA replication. Gene.

[33] Armstrong L, Arrington A, Han J, Gavrikova T, Brown E, Yamamoto M, Vickers SM, Davydova J (2012). Generation of a novel, cyclooxygenase-2-targeted, interferon-expressing, conditionally replicative adenovirus for pancreatic cancer therapy. Am J Surg.

[34] Chen RF, Lee CY (2014). Adenoviruses types, cell receptors and local innate cytokines in adenovirus infection. Int Rev Immunol.

