# Supplementary figures and images for: Cytotoxicity of replication-competent adenoviruses powered by an exogenous regulatory region is not linearly correlated with the viral infectivity/gene expression or with the E1A-activating ability but is associated with the p53 genotypes
Source: BMC Cancer. 2017 Sep 5;17:622. doi: 10.1186/s12885-017-3621-x (PMC5584036; doi:10.1186/s12885-017-3621-x)

Supplementary Figure 1

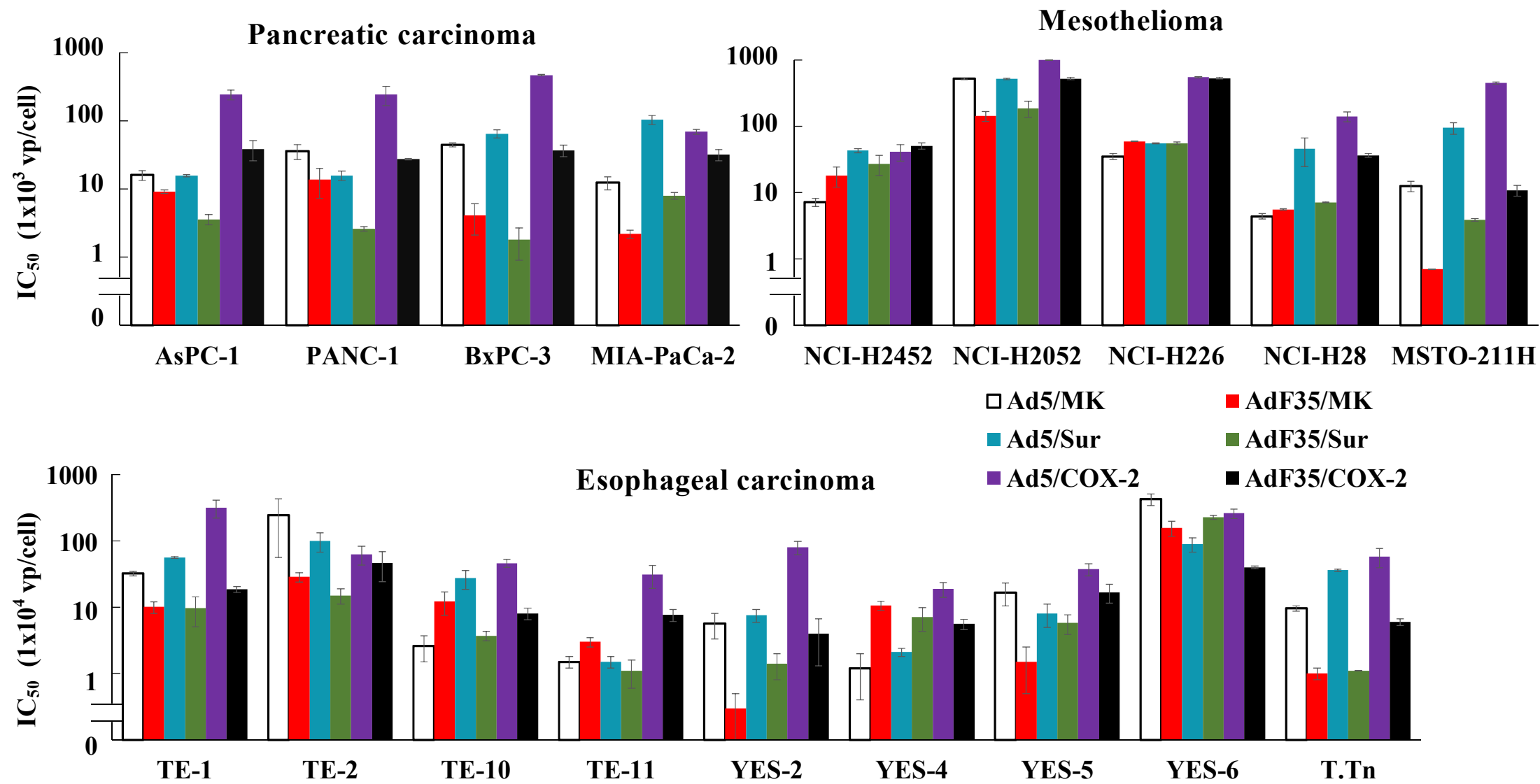

Supplement: Additional file 1: Figure. S1. — Cytotoxicity of replication-competent Ad on carcinoma cells. The same data in Table 1 were used. Averages and SEs are shown (n = 3) (PDF 128 kb) [file 12885_2017_3621_MOESM1_ESM.pdf]

Supplementary Figure 2

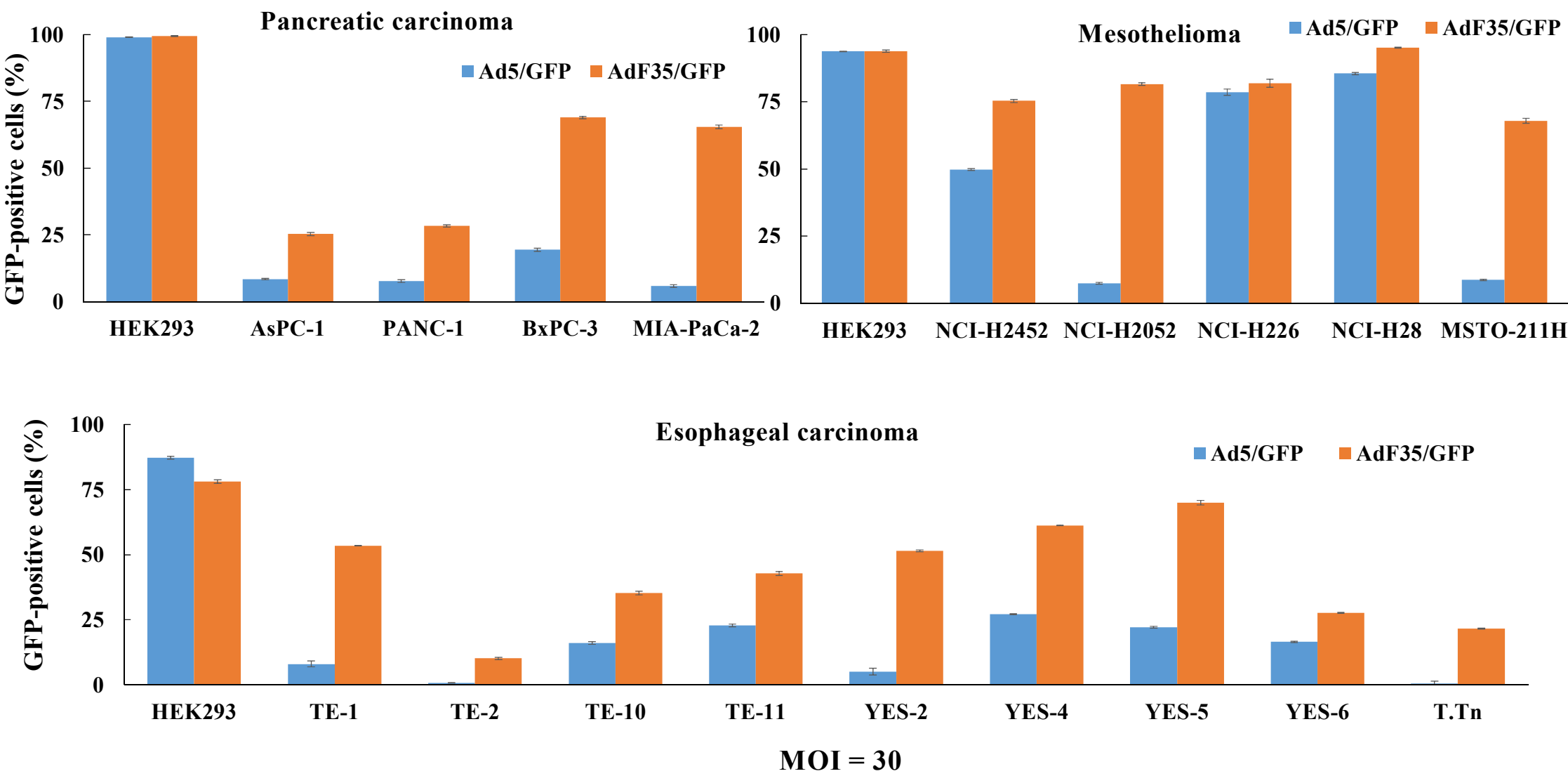

Supplement: Additional file 2: Figure. S2. — Infectivity/gene expression of Ad5/GFP and AdF35/GFP. Cells infected with Ad5/GFP or AdF35/GFP at 30 MOI were analyzed for the fluorescence intensity with flow cytometry. The same data in Table 2 were used. Averages and SEs are shown (n = 3) (PDF 121 kb) [file 12885_2017_3621_MOESM2_ESM.pdf]

Supplementary Figure 4

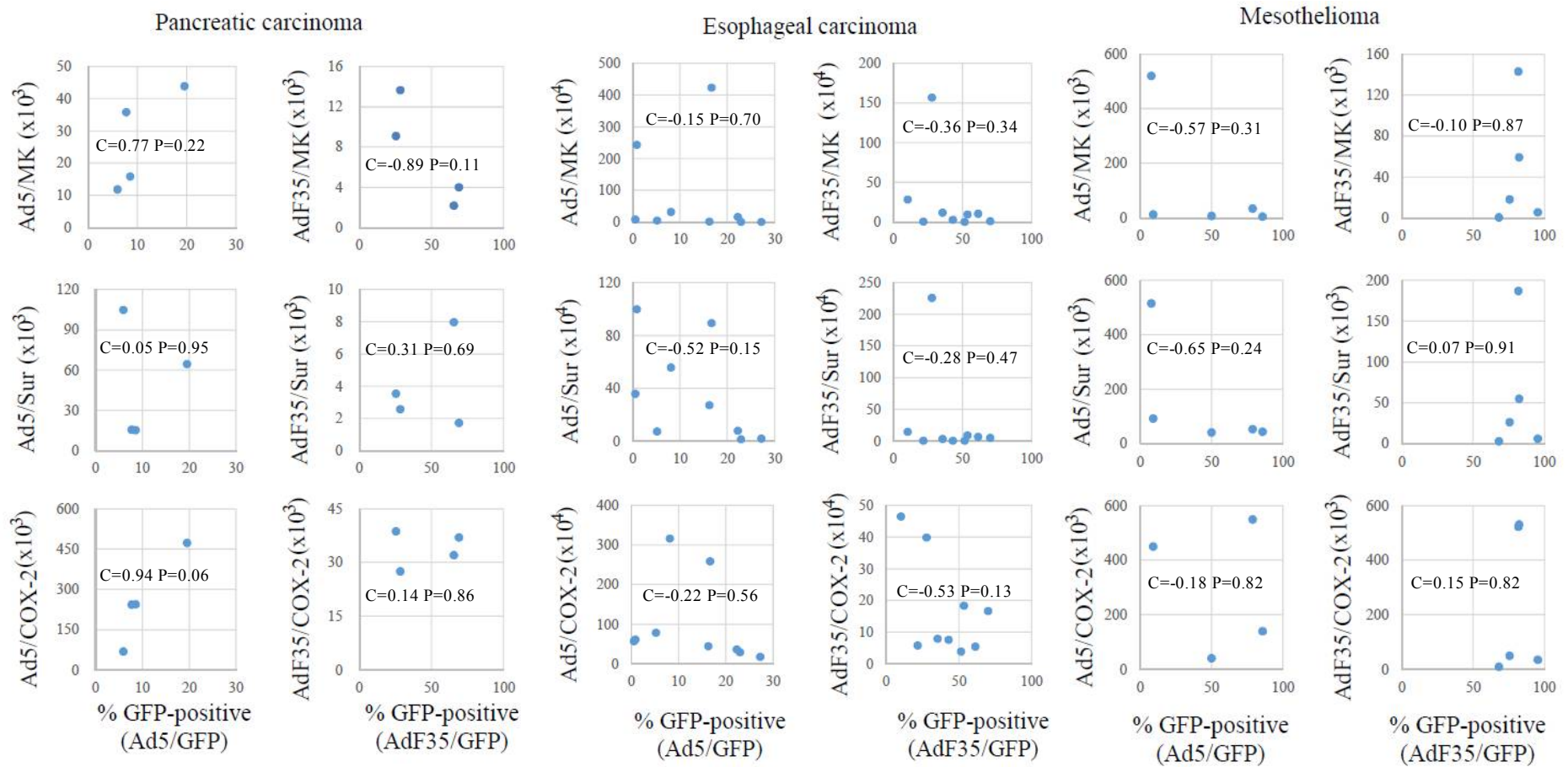

Supplement: Additional file 4: Figure S4. — Individual data of Ad-mediated cytotoxicity and percent GFP-positive cells in respective cells. The summary of correlation coefficient is shown in Table 4. Correlation coefficient (C) and P value are also shown (PDF 173 kb) [file 12885_2017_3621_MOESM4_ESM.pdf]

Supplementary Figure 5

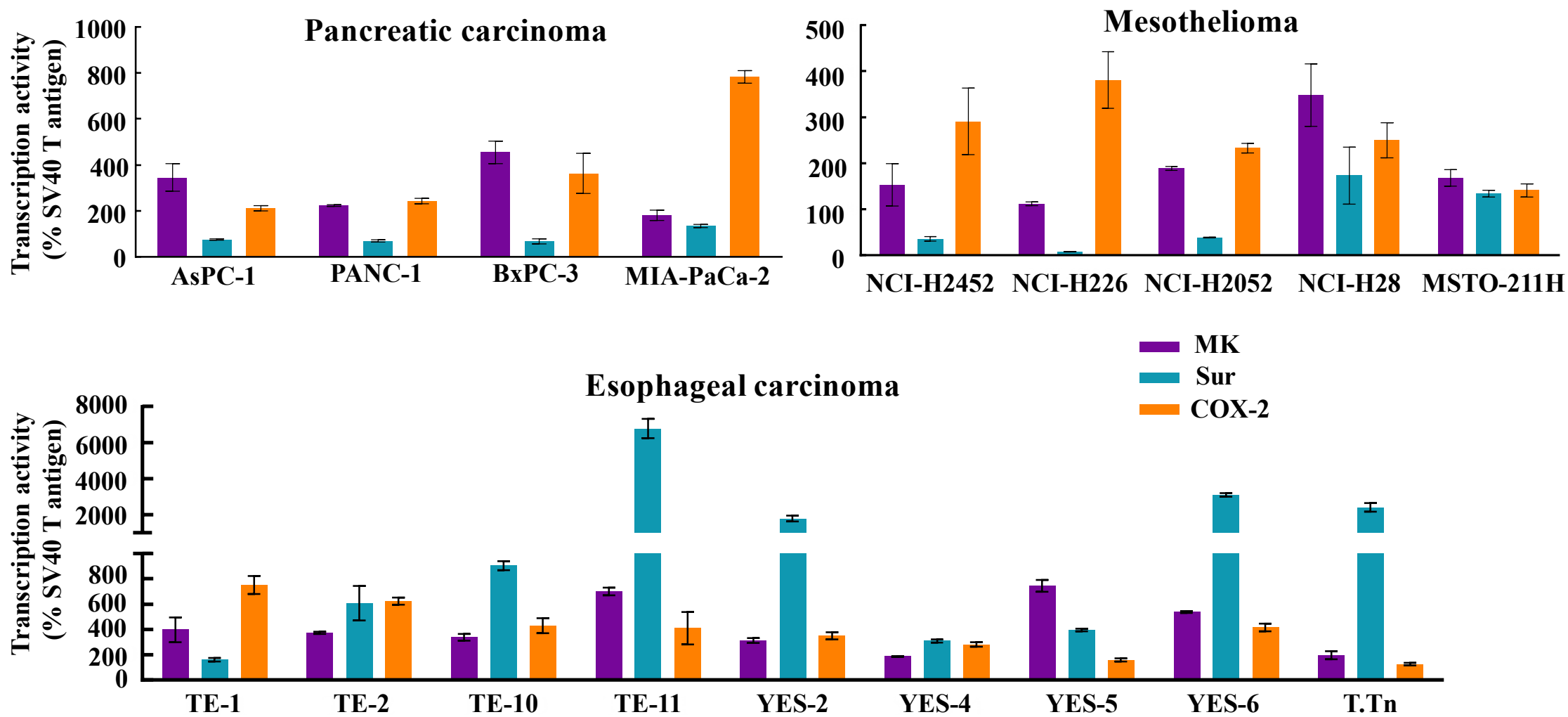

Supplement: Additional file 5: Figure S5. — Transcriptional activity of the regulatory region in target cells. The same data in Table 5 were used. Averages and SEs are shown (n = 3) (PDF 155 kb) [file 12885_2017_3621_MOESM5_ESM.pdf]

Supplementary Figure 6

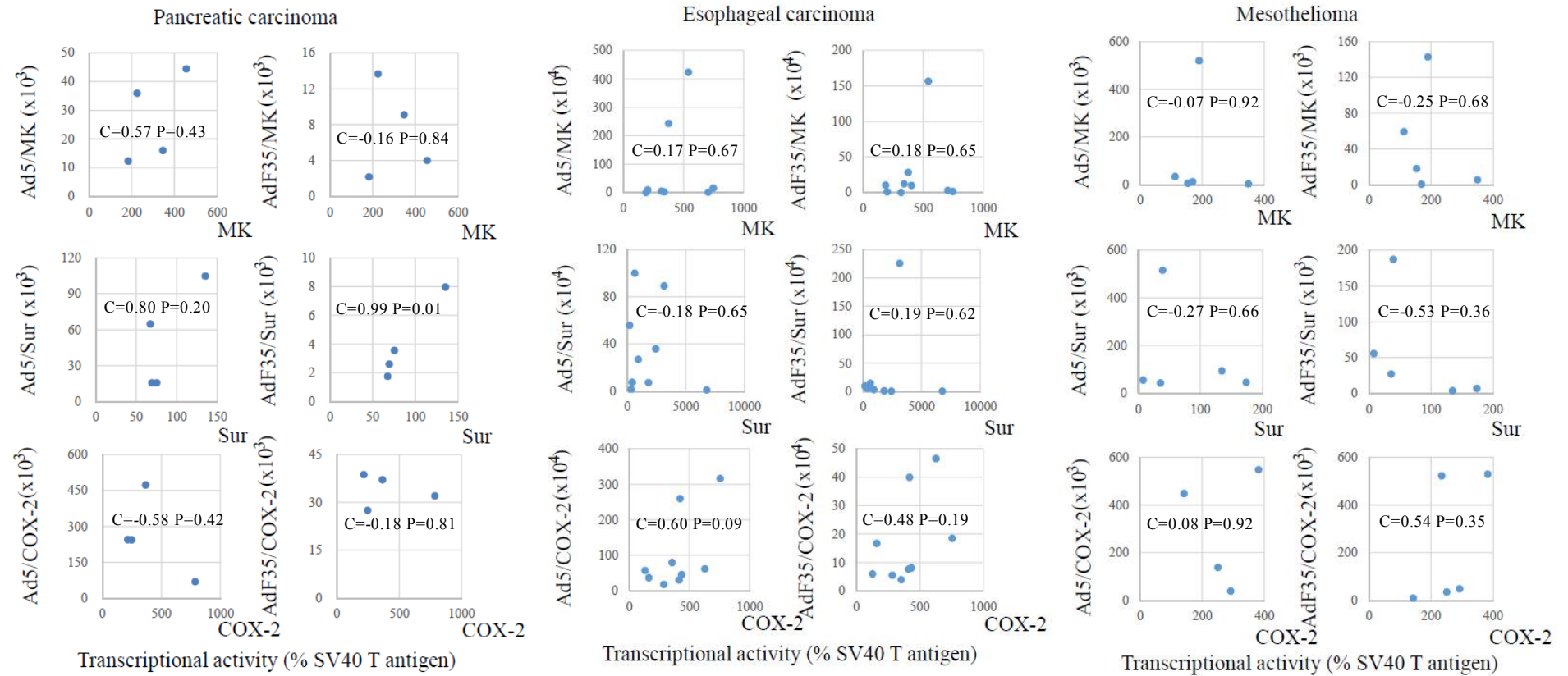

Supplement: Additional file 6: Figure S6. — Individual data of Ad-mediated cytotoxicity and transcriptional activity in respective cells. The summary of correlation coefficient is shown in Table 6. Correlation coefficient (C) and P value are also shown (PDF 177 kb) [file 12885_2017_3621_MOESM6_ESM.pdf]

Supplementary Figure 7

p53

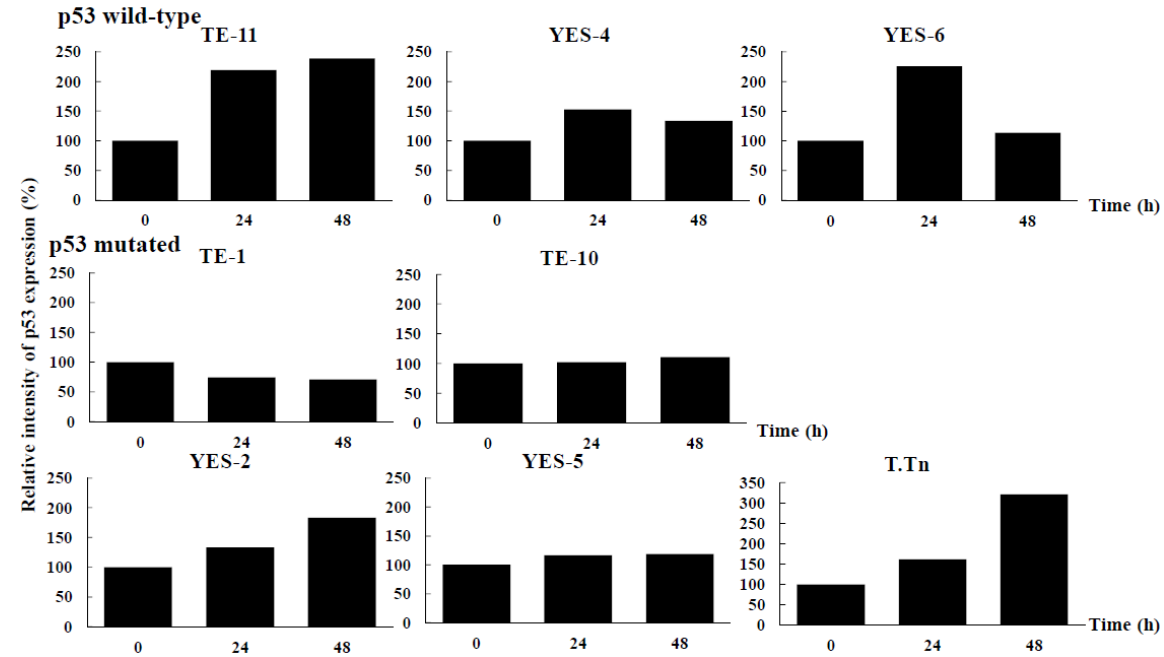

p21

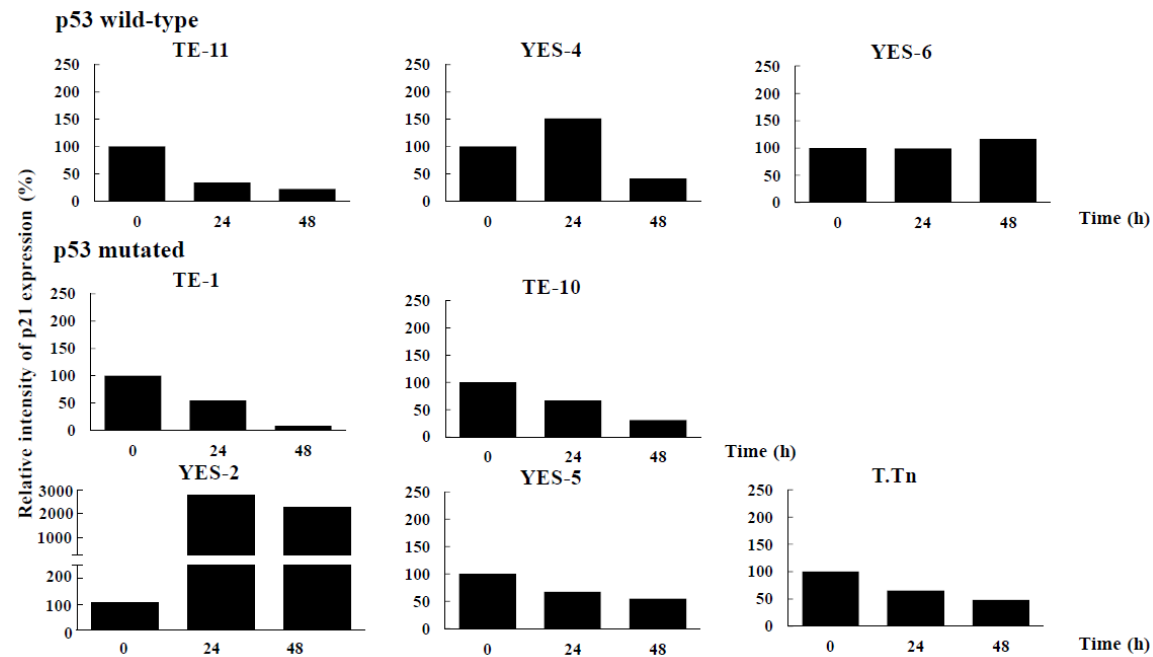

Supplement: Additional file 7: Figure S7. — Quantification of p53 and p21 expression of Fig. 2. Intensity of respective bands were measured with an image analyzer software, ImageJ (https://imagej.nih.gov/ij/). Relative intensity of p53 and p21 was adjusted by actin intensity and shown as a percentage of cisplatin (CDDP)-untreated cells. Expression of p53 and p21 in TE-2 cells was almost undetectable and the intensity data were not included (PDF 131 kb) [file 12885_2017_3621_MOESM7_ESM.pdf]

Supplementary Figure 8

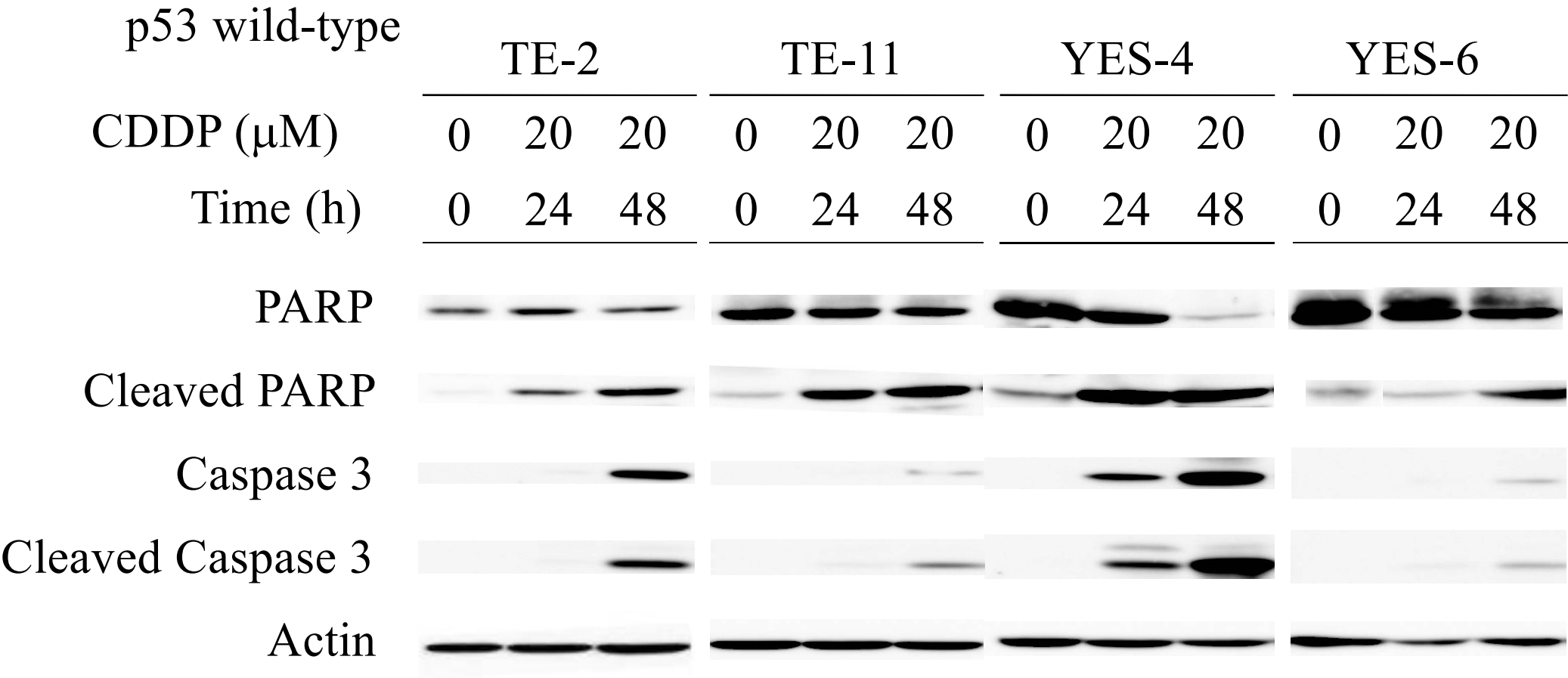

Supplement: Additional file 8: Figure S8. — Cisplatin (CDDP) induced cleavages of caspase-3 and PARP. Esophageal carcinoma cells with the wild-type p53 genotype were treated with CDDP as shown and the cell lysate was probed with the antibody as indicated, poly (ADP-ribose) polymerase (PARP) (can also detect cleaved PARP, #9542), and cleaved caspase-3 (can also detect caspase-3, #9661) (Cell Signaling). Actin was used as a loading control and the blot was the same as that in Fig. 2. Data of untreated YES-6 cells were taken in the same blot as other data (YES-6 cells treated with CDDP), but the sample was loaded next to that of YES-6 cells treated with CDDP for 48 h. The data untreated YES-6 cells were therefore moved to before the data of YEST-6 cells treated with CDDP for 24 h (PDF 118 kb) [file 12885_2017_3621_MOESM8_ESM.pdf]
