# Supplementary material for: Cytotoxicity of replication-competent adenoviruses powered by an exogenous regulatory region is not linearly correlated with the viral infectivity/gene expression or with the E1A-activating ability but is associated with the p53 genotypes
Source: BMC Cancer. 2017 Sep 5;17:622. doi: 10.1186/s12885-017-3621-x (PMC5584036; doi:10.1186/s12885-017-3621-x)

Supplementary Figure 3

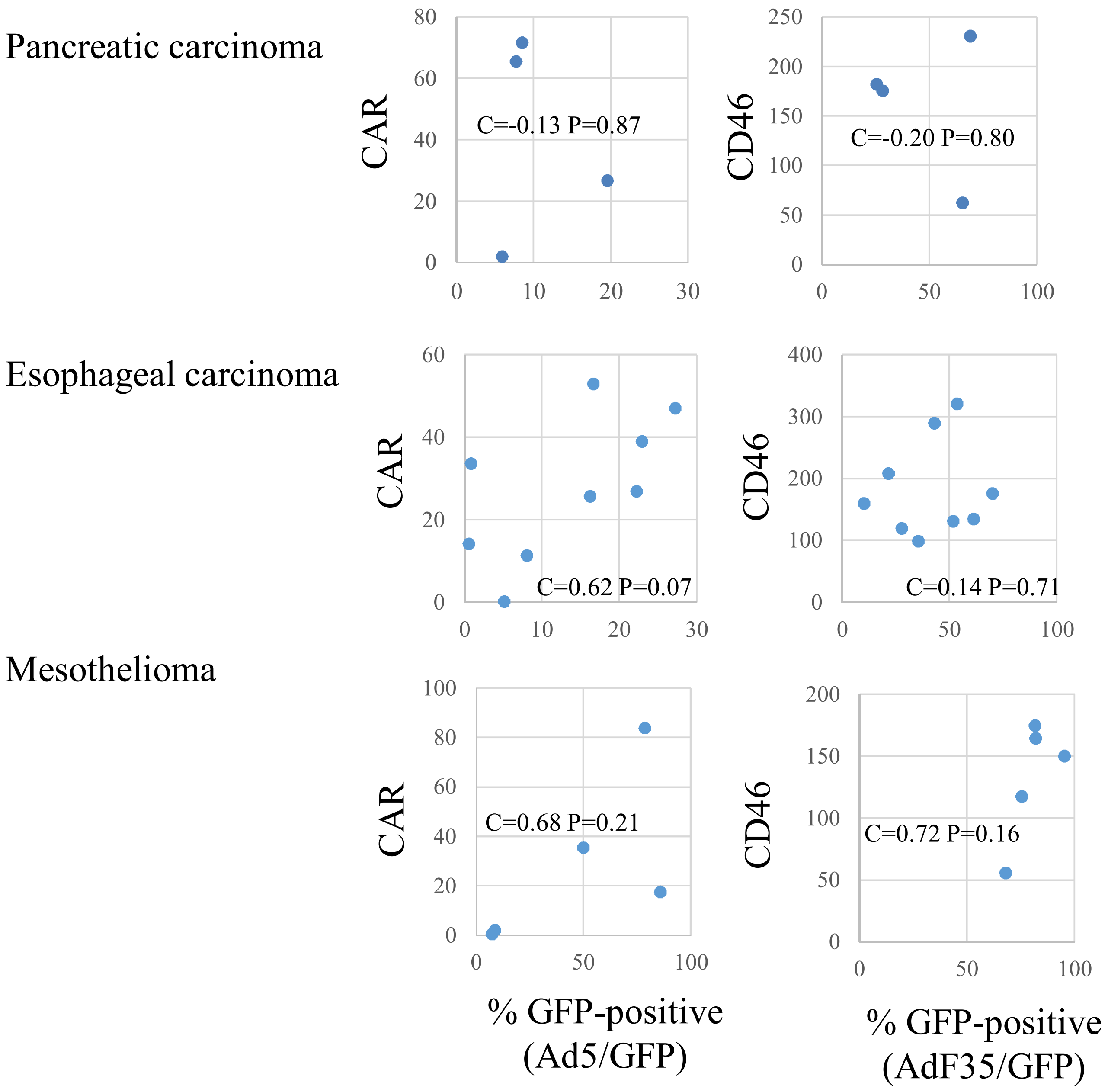

Supplement: Additional file 3: Figure S3. — Individual data of CAR or CD46 expression levels and percent GFP-positive cells in respective cells. The summary of correlation coefficient is shown in Table 3. Correlation coefficient (C) and P value are also shown (PDF 79 kb) [file 12885_2017_3621_MOESM3_ESM.pdf]
